# Effects of the Addition of Amino-Terminated Highly Branched Polyurea on Curing Properties of Phenol-Formaldehyde Resin

**DOI:** 10.3390/ma16103620

**Published:** 2023-05-09

**Authors:** Shiguan Lian, Huali Lin, Wenbin Zhang, Hong Lei, Ming Cao, Jianrong Mao, Taohong Li, Shi Chen, Long Yang

**Affiliations:** 1 Yunnan Provincial Key Laboratory of Wood Adhesives and Glued Products, Southwest Forestry University, Kunming 650224, China; 2 International Joint Research Center for Biomass Material, Ministry of Science and Technology, Southwest Forestry University, Kunming 650224, China; 3 College of Chemistry and Material Engineering, Zhejiang Agriculture and Forestry University, Hangzhou 311300, China

**Keywords:** phenol-formaldehyde (PF) resin, highly branched polyurea (HBP-NH_2_), adhesives, curing performance

## Abstract

In this work, a highly branched polyurea (HBP-NH_2_) similar to urea structure was introduced to phenol-formaldehyde (PF) resin to accelerate itscuring speed The results of gel time and bonding strength were combined to obtain a good modified additional stage and amount of HBP-NH_2_. The relative molar mass changes of HBP-NH_2_-modified PF resin were investigated by gel permeation chromatography (GPC). The effects of HBP-NH_2_ on the curing of PF resin were investigated by differential scanning calorimetry (DSC) and dynamic mechanical analysis (DMA). The effect of HBP-NH_2_ on the structure of PF resin was also investigated by nuclear magnetic resonance carbon spectroscopy (^13^C-NMR). The test results show that the gel time of the modified PF resin was reduced by 32% and 51% at 110 °C and 130 °C, respectively. Meanwhile, the addition of HBP-NH_2_ increased the relative molar mass of PF resin. The bonding strength test showed that the bonding strength of modified PF resin increased by 22% after soaking in boiling water (93 °C ± 2) for 3 h. The DSC and DMA analysis indicated that the curing peak temperature decreased from 137 °C to 102 °C, and the curing rate of the modified PF resin was also faster than that of the pure PF resin. The ^13^C-NMR results showed that HBP-NH_2_ in the PF resin reacted to produce a co-condensation structure. Finally, the possible reaction mechanism of HBP-NH_2_ for the modification of PF resin was given.

## 1. Introduction

Phenol-formaldehyde (PF) resin is widely used in outdoor building materials due to its high bonding strength and excellent chemical durability. However, PF resin exhibited slow curing rate, a dark color, and high brittleness after curing in the wood industry. Therefore, to accelerate the curing rate of PF resins, much attention is being paid to its low production efficiency and high energy consumption caused by its relatively slow curing rate [1,2].

A lot of modification research works have been done on the curing behavior of PF resin [3]. For example, urea [4,5,6,7], divalent metal ions [8,9,10,11], esters or carbonates [12,13,14,15,16], and biomass material [17,18,19]. Interestingly, urea can serve as an idea candidate due to its low cost to improve the curing properties of PF resin. The mechanism of urea-modified PF resins has been explained as well. Urea or phenol can react with formaldehyde, respectively, to produce hydroxymethyl compounds, and then can be co-condensed with free phenol or urea to produce phenol-urea-formaldehyde resin [6,20,21,22,23,24]. However, under alkaline conditions, the polycondensation process of PUF resin was relatively slower than that under acid conditions [20,21]. In addition, urea was added to PF resin with a high amount, the formation of co-condensed methylene structure was accompanied by the formation of small molecules of substituted urea. Absence of free urea or urea-derived small molecule structure in the PUF polycondensation could lead to a decrease in the mechanical strength of wood-based panels [25,26]. Therefore, a compound with terminal amino group being similar to urea and a larger molecular weight is found, and it may be used to improve the curing properties of PF resin without deteriorating itsbonding strength.

During the past decades, highly branched polymers were studied due to their attractive properties, such as low viscosity, good solubility, and high functionality, etc. It is reported that highly branched polymers can enhance the toughness and mechanical strength of thermosetting resins.It is found that highly branched polymers can improve their curing properties as well [27,28,29].

**Figure 1:**
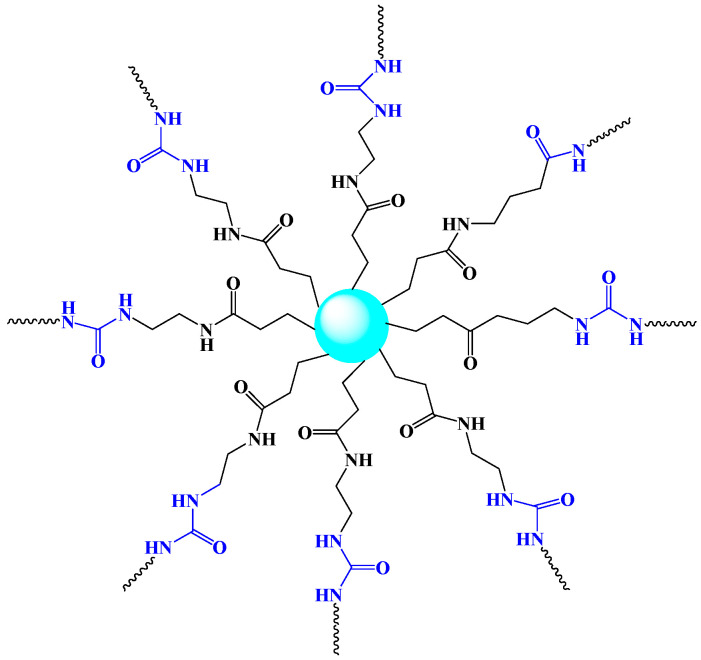
The structure of HBP-NH_2_.

Recently, a class of highly branched polyurea (HBP-NH_2_) prepared by triethylenetetramine and urea was synthesized, the structure is shown in Figure 1 [30]. The polyurea combines the advantages of urea and highly branched polymers. Furthermore, it has a highly branched structure, high molecular weight, good water solubility, and urea-like reactivity. Its end group is structurally similar to that of urea, and it has more reactive sites than urea. Therefore, it shows a higher reactivity to formaldehyde and methylol phenol than monomer urea. This is beneficial for its polycondensation with PF resin. A highly branched and cross-linked structure is one of the key points for its good bonding performance as a thermosetting wood adhesive resin. Additionally, the highly branched structure and high molecular weight of HBP-NH_2_ are more conducive to generate a three-dimensional network structure so that the curing of PF resin can be improved.

Inspired by the condensation reaction between urea and PF resin, HBP-NH_2_ was utilized to modify phenol-formaldehyde resin to react with formaldehyde and methylol phenol. The effect on the curing properties of phenolic resin by HBP-NH_2_ addition was examined. Gel time, DSC, and DMA were adopted to analyze the curing rate of modified phenolic resins for obtaining the optimal process of HBP-NH_2_-modified phenolic resins. The relative molecular mass of PF resins before and after modified were also tested using GPC to investigate the relationship between molecular weight and curing rate. The bonding strength of plywood sample bonded by PF- and HBP-NH_2_-modified PF resin was evaluated. The co-condensation reactions between HBP-NH_2_ and PF were investigated by ^13^C-NMR, and the mechanism of PF modification with HBP-NH_2_ was then proposed.

## 2. Materials and Methods

### 2.1. Materials

Phenol, formaldehyde (aqueous solution, 37 wt%), urea, triethylenetetramine, methyl acrylate, ethylenediamine, and sodium hydroxide were obtained from Sinopharm Chemical Reagent Co., Ltd. in Shanghai, China. All chemicals in this research were used directly without any purification.

### 2.2. Synthesis of HBP-NH_2_

HBP-NH_2_ was prepared by following the process mentioned in the literature [30]. Briefly, anhydrous methanol was added to a flask as solvent in an ice bath. Then triethylenetetramine was added and stirred well. Methyl acrylate was added to the mixture through a constant pressure funnel, and then the mixture was brought to room temperature. The reaction was performed at room temperature for 24 h under magnetic stirring. Thereafter, the solvent anhydrous methanol and excess methyl acrylate were removed under vacuum. The solution named as HBP1 was obtained. Then, certain amount of anhydrous methanol was introduced into the HBP1 solution and stirred thoroughly. Ethylenediamine was added to the as-obtained mixture slowly at room temperature by a constant pressure drop funnel. The reaction was stirred at room temperature for 48 h more. After that, methanol and excess ethylenediamine were removed by distillation under reduced pressure yielding the product named as HBP2. Urea was added to HBP2 and mixed in a flask with a molar ratio of urea/HBP2 of 5.0. The mixture was heated to 120 °C in an oil bath by mechanically stirring for 10 h to obtain HBP-NH_2_. The resultant HBP-NH_2_ is a yellow solid, which is prepared as 50% aqueous solution before utilization.

### 2.3. Synthesis of PF Resin and Modified PF Resins

**Figure 2:**
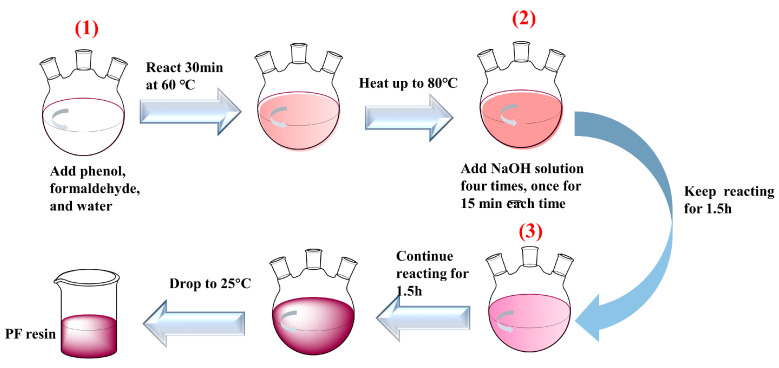
The synthesis of PF resin.

The PF resin was synthesized according to the molar ratio of phenol to formaldehyde of 1:2.1. The synthesis process is shown in Figure 2. First, a certain mass of phenol, formaldehyde, and distilled water (27–29% of the mass of phenol) was sequentially added to a three-necked flask. Then, it was placed in a water bath at 60 °C for 30 min under magnetic stirring, and a quarter of NaOH solution (30 wt%) was added, and another quarter of NaOH solution was added every 15 min. The molar ratio of total sodium hydroxide to phenol is 0.2:1. Finally, the mixture was continued to be stirred at 80 °C for 3 h to obtain PF resin.

HBP-NH_2_ was added at different stages of the phenolic resin synthesis process to obtain modified PF resin, named PFA, PFB, and PFC, respectively. HBP-NH_2_ was added at 1%, 3%, 5%, 7%, 9%, and 11% of the mass of PF solution, respectively. As shown in Figure 2, PFA was synthesized by adding HBP-NH_2_ at the end of the phase (1). The resins obtained with different additions of HBP-NH_2_ were marked as PFA1, PFA3, PFA5, PFA7, PFA9, PFA11, respectively. PFB was synthesized by adding HBP-NH_2_ at the end of the phase (2). The resins obtained with different additions of HBP-NH_2_ were marked as PFB1, PFB3, PFB5, PFB7, PFB9, PFB11, respectively. PFC was synthesized by adding HBP-NH_2_ at the end of phase (3). The resins obtained with different additions of HBP-NH_2_ were marked as PFC1, PFC3, PFC5, PFC7, PFC9, PFC11, respectively.

### 2.4. Measurements of the Basic Properties of the Resins

After synthesis, solid content and viscosity of the resins were measured. In solid content measurement, 1.0 g liquid resin was poured into an aluminum dish and then placed in a drying oven at 120 ± 1 °C for 2 h. The solid content was calculated by the percentage of the weight of nonvolatile substances to liquid resin. The average value of three replications was presented. The viscosities of the resins were measured at 25 °C, using an SDB-2 rotary viscometer (QINGHUA Instruments, Shanghai, China).

### 2.5. Gel Time Determination

The gel time determination of PF resin was based on the Chinese national standard [31] (GB/T 33315-2016). About 0.5 mL of PF resin was added to the test tube, and the test tube was immersed in an oil bath (130 °C and 110 °C, respectively) for testing. The time required for the adhesive to appear in gelation was defined as the gel time. The manual method was used and the average value of three replications was presented.

### 2.6. Gel Permeation Chromatography (GPC)

Gel permeation chromatography (GPC) was performed on a PL-GPC50 gel permeation chromatograph equipped with an aqua gel-OHMixed-M gel column, and the column temperature was maintained at 40 °C. Detection was carried out on a differential refractive index detector, deionized water (0.1 M sodium nitrate) was used as the eluent, the flow rate was 1.0 mL/min, and the injection volume was 100 µL. Calibration curves were determined from a series of narrowly distributed polyethylene oxide (PEO) standards and were used to calculate the relative molar masses of the samples.

### 2.7. Differential Scanning Calorimetry (DSC)

A 5 mg sample of PF resin was placed in a 204F1 DSC instrument (Perkin-Elmer, Rodgau, Germany) for curing temperature testing. The protective gas was N_2_, the heating rate was 10 °C/min, and the temperature range of the PF resin test was 30–250 °C. The software used for DSC data treatment was PYRISTM Version 4.0.

### 2.8. Dynamic Thermomechanical Analysis (DMA)

The curing rates of the PF resins were processed using a dynamic thermomechanical analysis (DMA) instrument (DMA242E, NETZSCH Company, Germany) and software Proteus Version 4.2 (DMA 242 EArtemis). The liquid PF resin (150 mg) was spread onto two veneers with the size of 50 mm × 10 mm. Two veneers were placed in stacks, and performed on universal mechanical equipment. The temperature range was 35 °C to 250 °C, and the heating rate was 5 °C/min.

### 2.9. ^13^C Nuclear Magnetic Resonance (^13^C-NMR)

The ^13^C nuclear magnetic resonance (^13^C-NMR) spectra were measured using a Bruker AVANCE NEO 500 spectrometer (Bruker Corporation, Zurich, Switzerland). The ^13^C-NMR samples were prepared by dissolving 300 µL of samples in 100 µL of acetone-d6. The spectra were recorded with a pulse angle of 90 degrees (12 µs) and a relaxation delay of 2 s. The spectra were taken at 150 MHz with 400 accumulated scans.

### 2.10. Preparation of Three-Layer Plywood Panels and Bonding Performance Tests

**Figure 3:**
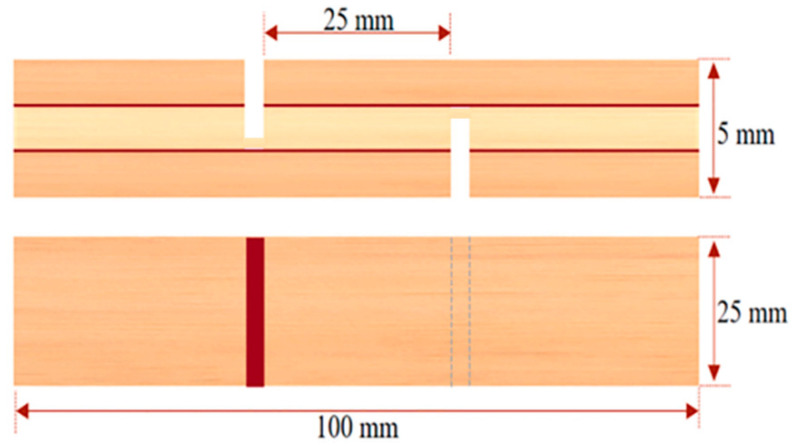
Specimen size diagram.

Three-layer plywood panels were prepared using the PF and modified PF resins with poplar veneers in size of 180 mm × 110 mm × 4.5 mm. Three-layer plywood panels were prepared by spreading the adhesive on double sides of the central layer in 300 g/m^2^. After resting at room temperature for 15 min, the assembled plywood panels were hot-pressed at 140 °C for 5 min under pressure of 0.7 MPa. The prepared plywood was stored at room temperature for 24 h and then each panel was cut into six samples. Then each specimen was slotted according to China National standard [32] GB/T 9846-2015 to obtain a specimen with shearing (gluing) area of 25 mm × 25 mm. The specimen size is shown in Figure 3.

To evaluate the water resistance of the resins, wet shearing strength tests were carried out. After soaking the sample in 93 °C boiling water for 3 h, they were cooled to room temperature and then the adhesive strength was tested on a WDS-50KN machine (Shimadzu, Kyoto, Japan), and calculated using the following formula: shearing strength (MPa) = maximum force (N)/gluing area (mm^2^).

## 3. Results and Discussion

### 3.1. The Basic Properties of PF and HBP-NH_2_ Modified PF Resins

**Figure 4:**
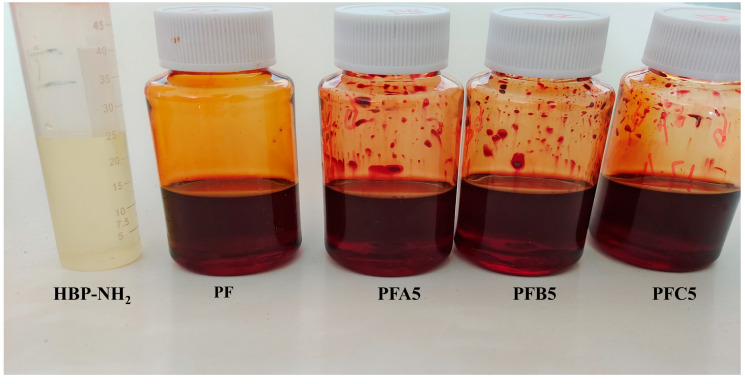
Sample of HBP-NH_2_ and PF resin.

The images of HBP-NH_2_ and PF resin samples are shown in Figure 4. The apparent color for HBP-NH_2_ modification did not change remarkably, showing a dark brown color. The physical property parameters of PF resin and modified PF resins are shown in Table 1. As shown in the Table 1, the solid content before and after modification for a series of PF resins is between 44% and 48%. This was due to the addition of HBP-NH_2_ as a 50% solution and the low addition level, thus there was almost no effect on the solid content of the PF resins. The viscosity of the modified PF resins can be observed in Table 1, and the viscosity increased by addition of HBP-NH_2_. It was possible that the crosslink density of the synthesized modified PF resin increased leading to an increase in viscosity by introducing HBP-NH_2_. In addition, the viscosity of PFB resin was greater than that of PFA and PFC resins even with the addition of equal amounts of HBP-NH_2_. This phenomenon was attributed to co-condensation between HBP-NH_2_ and hydroxymethyl phenol formed in the system during the reaction, as the addition of HBP-NH_2_ prior to sodium hydroxide. The molecular weight of the PFB resin went up further resulting in an increased viscosity. The viscosity for PFC resin showed a lower value, smaller than the pure PF resin. HBP-NH_2_ was added after the phenol polymer was formed from phenol and formaldehyde, and as HBP-NH_2_ has more active sites and is not fully bound, it resulted in a large relative molecular mass to complete the gel curing. Therefore, its crosslink density is relatively lower, the liquid is more fluid, and its viscosity is relatively lower. When HBP-NH_2_ addition exceeded 5%, the viscosity of PFC increased significantly which was related to polymerization and crosslink density of the resins. Notably, the modified PF resins maintained some fluidity after storing for 60 days. Andthe pure PF resin had cured. It indicates that HBP-NH_2_ is beneficial to prolong the pot-life of PF resin.

The gel time is generally defined as the conversion time required for a prepolymer to transform from a disordered liquid to a three-dimensional macromolecular structure under specific conditions and can be used as an indicator of the curing speed and molecular reactivity of PF resins [33,34]. The gel time for pure PF resin was 592 s under test conditions at 110 °C. The gel time of PFA1, PFB1, and PFC1 resins was lower than that of pure PF resin without any remarkable distinction. This result was obtained owing to the low content of HBP-NH_2_ and also the slower intermolecular motion at 110 °C, which reduces collisions between molecules, resulting in a slower curing rate. The curing rate of modified PF resin increased significantly but still less than 500 s when the HBP-NH_2_ addition was higher than 1%. Meanwhile, the gel time shortened with the increase in HBP-NH_2_ addition. The gel time of the modified PF resin was shorter than the same set of samples when the HBP-NH_2_ addition was about 5%. When the HBP-NH_2_ addition amount continued to increase, the gel time increased. This was owing to the fact that HBP-NH_2_ was more completely condensed with hydroxymethyl phenol in the PF resin system when the amount of addition was about 5%. With the continued addition of HBP-NH_2_, the co-condensation reaction with PF resin no longer continued. The excess HBP-NH_2_ underwent self-condensation polymerization. According to the work of Yang [30], self-condensation between HBP-NH_2_ requires a higher temperature. Thus, the HBP-NH_2_ addition reduced the curing rate of modified PF resins at 110 °C. As the temperature increased from 110 °C to 130 °C, the gel time for the modified PF resins significantly decreased. This is because the molecular chains could move faster at high temperature and intermolecular bonding was easier [35]. In addition, the same trend of the modified resin was obtained at 130 °C compared to at 110 °C. Significantly, the gel time of the control PF resin was 445 s at 130 °C, but for the modified resins it was lower than 445 s even at 110 °C. It was explained that a reasonably controlled addition conditions of HBP-NH_2_ could reduce the curing temperature of the resin. In summary, the addition of HBP-NH_2_ to PF resin could promote its faster curing. The curing rate of PF resins was accelerated with the increase in the amount of HBP-NH_2_. When the amount of HBP-NH_2_ is near 5%, the curing rate of the modified resin increasedsignificantly.

In addition, the gel times exhibited by the addition of HBP-NH_2_ at different stages of PF resin synthesis differed at an identical horizontal gradient. The gel time for PFC resin is shorter than that of PFB resin, while for PFA resin is relatively longer. The possible reason was that when HBP-NH_2_ was added to the PFC resin at 1.5 h near the end of the synthesis, larger molecular weight phenolic self-condensation products were formed in the PF resin system, and the HBP-NH_2_ could cross-link these molecules again. The synthesis of PFB resin could be understood as the hydroxymethylation of phenol and formaldehyde first to produce hydroxymethyl phenol. The HBP-NH_2_ was hydroxymethylated by formaldehyde first, when it was added into the reaction and finally co-condensed with hydroxymethyl phenol. It will take more time than the PFC resin to form a three-dimensional network during the curing process. It can be speculated that the addition of HBP-NH_2_ at the initial stage of phenol and formaldehyde polymerization results in the formation of only some small molecules for the synthesis of PFA resin, which will take longer to form a three-dimensional mesh structure during curing. That is one of the main reasons for the slow curing speed of PFA compared to that of PFB and PFC resins.

Overall, the results of the gel time experiment show that the addition of HBP-NH_2_ could effectively improve the curing of PF resin and reduce the gel time. Meanwhile, in PF synthesis, when HBP-NH_2_ was added before the end of the reaction and the addition amount was 3–5%, the obtained modified PF resin had a shorter gel time.

### 3.2. GPC Analysis of PF Resins

**Figure 5:**
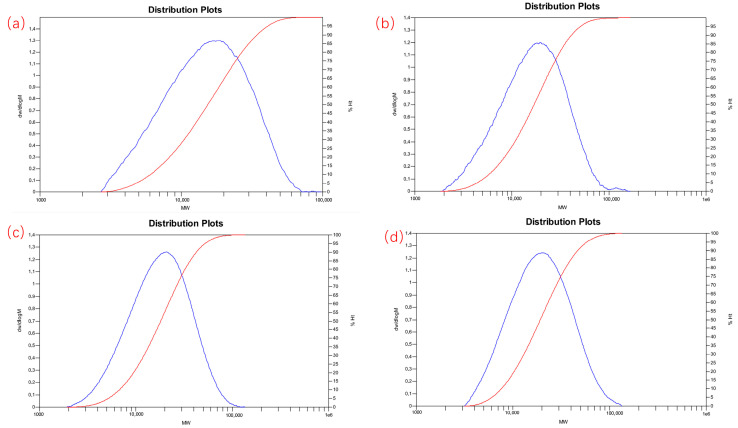
GPC curve results for phenol-formaldehyde resins. Note: (**a**) = PF resin, (**b**) = PFA5 resin, (**c**) = PFB5 resin, (**d**) = PFC5 resin. blue line = dw/dlogM, red line = Ht.

In order to clarify the effect of HBP-NH_2_ addition on the molecular weight of PF resin, GPC evaluation was conducted. The results are shown in Figure 5, and the main results are summarized in Table 2, including the number-average molecular mass (Mn), weight-average molecular mass (Mw), and polydispersity index (PDI) of the modified PF by HBP-NH_2_.

It was found that the addition of HBP-NH_2_ increased the Mn and Mw of the PF resin, resulting in a larger molar mass of the polymer. At the same time, HBP-NH_2_ was added later during the synthesis of PF resin, and the molar mass of PF resin was larger. The larger molar mass meant a faster curing rate as a wood adhesive, which was also reflected in the gel time measurement results. Similarly, the PDI of PF resin increased slightly with the addition of HBP-NH_2_. This may be caused by the condensation between multifunctional groups that occurred after the addition of HBP-NH_2_. However, the differences were not significant, all in the range of 1.5–2.0, indicating that the modified PF resin maintained good homogeneity [36,37]. Therefore, the addition of HBP-NH_2_, on the one hand, could increase the molar mass of PF resin, which could accelerate the curing rate. On the other hand, the modified PF resin still had good homogeneity, indicating that HBP-NH_2_, which possessed a highly branched structure, could generate a high density of crosslinking sites in the PF resin system [38]. In this sense, the addition of HBP-NH_2_ during PF resin synthesis can increase the molar mass while maintaining the high crosslink density of the resin. Perhaps the curing rate is accelerated without affecting the bonding properties.

### 3.3. Bonding Strength of Series PF Resins

**Figure 6:**
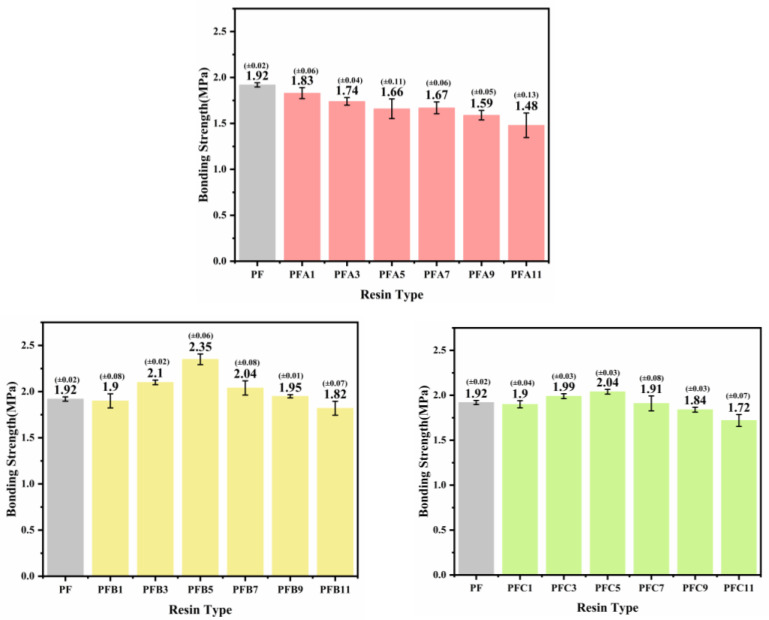
The bonding strength of PF, PFA, PFB, and PFC resins.

The gel time results showed that HBP-NH_2_ can accelerate the curing rate of the PF resin and reduce the curing temperature of it. As the amount of urea added increases, the curing rate of the urea-modified PF resin accelerates and the bonding performance decreases. Therefore, the bonding performance of HBP-NH_2_ modified PF resins were investigated in this research as well. After soaked in boiling water for 3 h, the bonding strengths of the modified PF resins were obtained and shown in Figure 6. The calculated coefficients of variation of all sample data were lower than 0.1, indicating that the results were normal and reliable. The bonding strength of pure PF resin was 1.92 MPa, showing a decrease in the synthesized PFA resins. A negative relationship was shown between the bonding strength and HBP-NH_2_ addition. This may be due to the reason that in the PF resin system, phenol and formaldehyde had not yet formed hydroxymethyl phenol, and the addition of HBP-NH_2_ would rob some of the formaldehyde, resulting in a decrease in the performance of the PFA resin. Meanwhile, with the increase in HBP-NH_2_ addition, the molar ratio of formaldehyde to phenol further decreased, resulting in a continuous decrease in bonding strength. When the amount of HBP-NH_2_ added was 1%, the bonding strength of PFB resin is consistent with that of pure PF resin. And the bonding strength was further strengthened as the amount added continued to increase. When the addition amount was 5%, the bonding strength increased by 22%. There is a possible reason. HBP-NH_2_ has a higher molecular weight and more terminal amino groups. After hydroxymethylation of phenol and formaldehyde, HBP-NH_2_ is added to synthesize PFB resin, which has a higher cross-linking density and density, thereby improving the bonding strength of PFB resin. But the bonding strength of PFB resin started to decrease when the addition amount of HBP-NH_2_ continued to increase. This is because excessive HBP-NH_2_ reacts with more formaldehyde, resulting in a decrease in the molar ratio of formaldehyde to phenol in the system. This leads to a decrease in the generation of hydroxymethyl phenol, which reduces the degree of condensation of PF resin, and thus reduces the bonding strength. Moreover, it could be seen from Figure 6 that the trend of the bonding strength of PFC resin was consistent with that of PFB. The difference was that the bonding strength of PFC resin was slightly lower than that of PFB resin. It may be due to the reason that the molecular chain of PF resin had been formed when HBP-NH_2_ was added. The crosslinking density of the prepared resin is lower than that of PFB resin. Although the bonding strength of PFB resin is slightly higher than that of PFC resin, PFC resin has an advantage in curing speed. Therefore, in order to balance the curing time and bonding strength, PFC resin can be preferred in practical applications. When 5% HBP-NH2 is added, the resin gel time is 226 s, and the bonding strength reaches 2.04 MPa.

### 3.4. DSC Analysis

**Figure 7:**
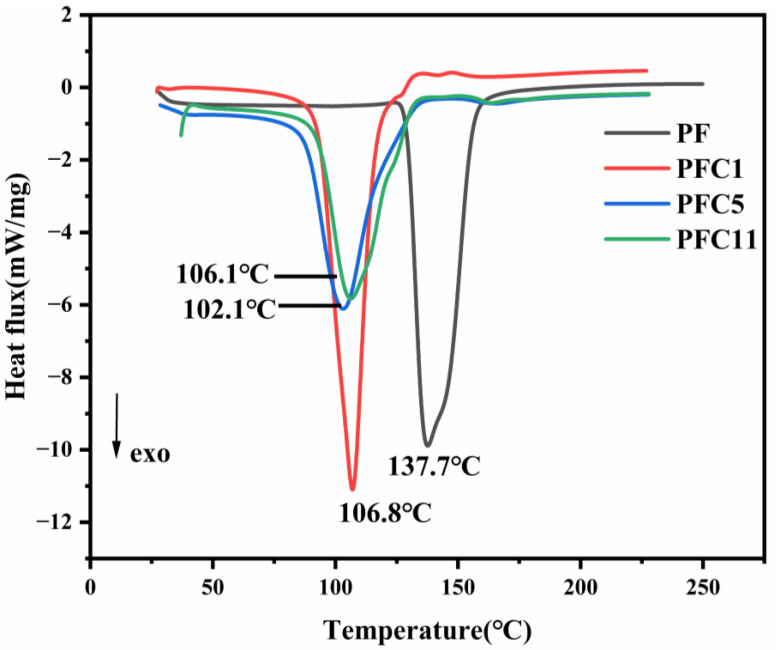
DSC curve diagram of PFC resins.

The gel time was determined as an early behavior of curing. Moreover, gelation is sometimes affected by the physical property of the resin, not completely dependent on polycondensation [38]. In order to further investigate the curing properties of the resin, the curing temperature of the modified PF resin was analyzed using DSC method. Because PFC resin has better gel time and bonding performance, this study only tested PFC resins by DSC. The effect of the addition of HBP-NH_2_ on the exothermic peak temperature in the curing reaction of PFC resin was studied. The results of the DSC tests are shown in Figure 7. The peak temperature of pure PF resin was 137.7 °C, and the peak temperatures of PFC1, PFC5, and PFC11 resins were 106.8 °C, 102.1 °C, and 106.1 °C, respectively. The peak temperatures of series PFC resins decreased with the increase in HBP-NH_2_ addition. When the addition amount of HBP-NH_2_ was 5%, the peak temperature was the lowest. When HBP-NH_2_ was continued to be added, the exothermic peak temperature increased instead. This test results were basically consistent with the gel time results. The exothermic peak temperatures of modified PF resins has been reduced by nearly 30 °C compared to pure phenolic resin. This indicates that HBP-NH_2_ can effectively reduce the curing temperature of PF resin. This means that modified PF resin as a wood-based panel adhesive requires a lower curing temperature, which has the potential to reduce production energy consumption in production. This may be similar to the mechanism of urea modified PF resin to reduce curing temperature. The HBP-NH_2_ added during the synthesis of PF resin has terminal amino groups similar to urea. The terminal amino group undergoes hydroxymethylation with formaldehyde, and then co-condensed with hydroxymethyl phenol, which reduced the curing temperature of PF resin [21]. Compared with urea, HBP-NH_2_ has high molecular weight, a highly branched structure, and more reactive sites. Therefore, during the curing process, it can quickly form a three-dimensional net structure, leading to rapid curing.

### 3.5. DMA Analysis

**Figure 8:**
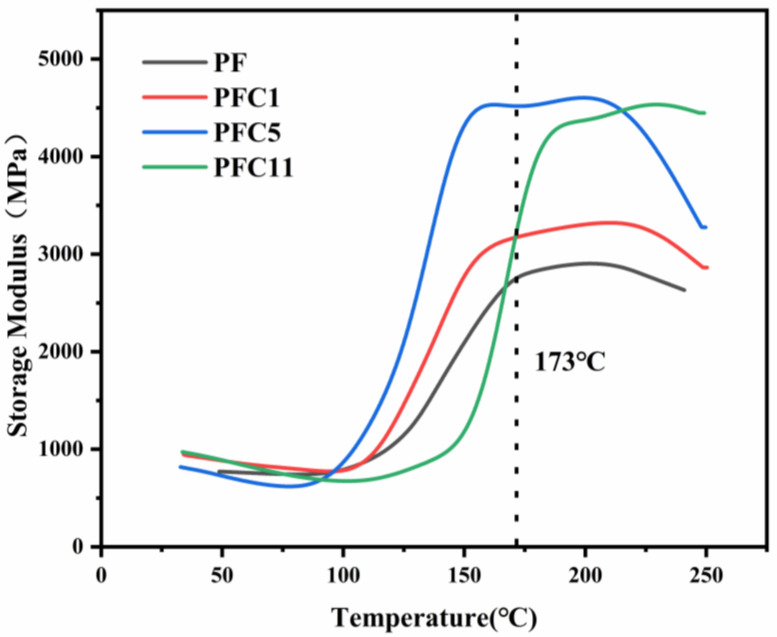
DMA curve diagram of PFC resins.

DMA could be used to study the curing properties of PF resins. The variation in the energy storage modulus of PFC resins with temperature is shown in Figure 8. The slope of the DMA curve could reflect the rate of condensation between resin molecules and could be used as a basis for the rate of resin curing reaction [39,40,41]. It could be seen from Figure 8 that the slopes of the DMA curves for PFC resins with HBP-NH_2_ addition were higher than that of pure PF resin, indicating that the addition of HBP-NH_2_ can promote the rapid curing of PF resin between wood strips.

When the resin reached the maximum modulus value, it indicated that the resin was fully cured and the condensation was complete [42,43]. As shown in Figure 8, the temperature at which the pure PF resin completed condensation was 173 °C. The PFC1 and PFC5 resins were basically below 173 °C, which was lower than the PF resin. While PFC11 resin was higher than 173 °C. It should be mentioned that HBP-NH_2_ itself can serve as a wood adhesive, but its curing temperature is close to 200 °C [30]. Therefore, when the addition of HBP-NH_2_ was too high, the curing of unreacted HBP-NH2 requires a higher temperature for self-curing, resulting in a higher curing temperature for the PF resin system.

### 3.6. ^13^C-NMR Analysis of PF Resins

**Figure 9:**
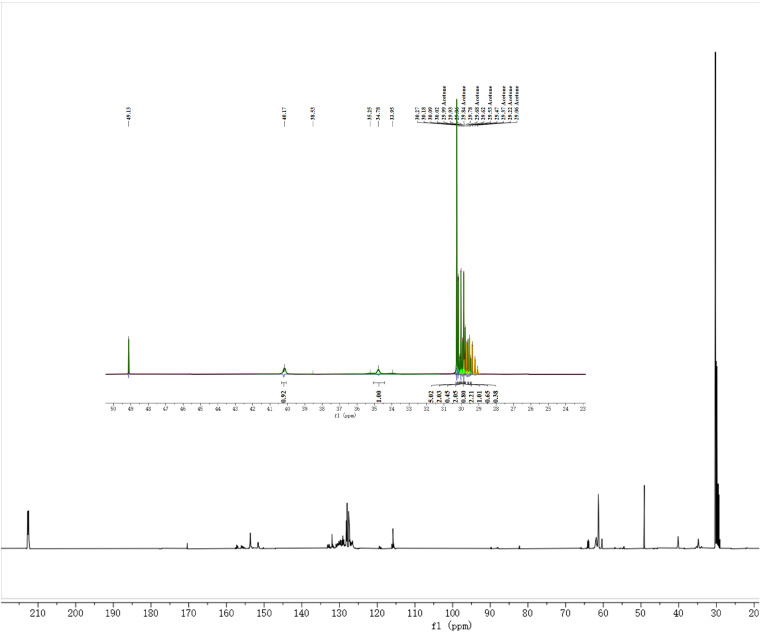
The ^13^C-NMR spectrum of PF resin. Note: The yellow one is the solvent peak, which belongs to the sevenfold peak of deuterated acetone. The green one is the test peak of the sample.

**Figure 10:**
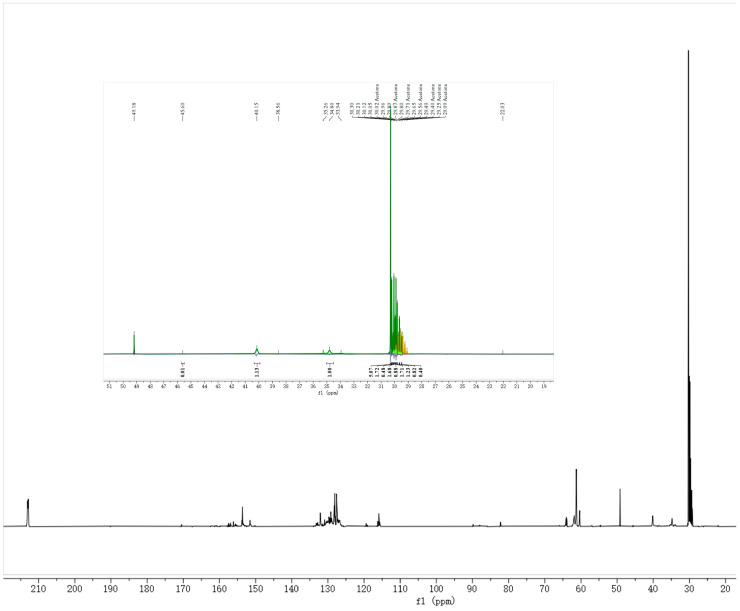
The ^13^C-NMR spectrum of PFC5 resin. Note: The yellow one is the solvent peak, which belongs to the sevenfold peak of deuterated acetone. The green one is the test peak of the sample.

The ^13^C-NMR spectra of PF and PFC5 resins are shown in Figure 9 and Figure 10, respectively. Based on the reaction mechanism of PUF, the PF resins were integrated and their ^13^C-NMR peaks were attributed as shown in Table 3 [44,45,46]. At 45–46 ppm, it can correspond to the co-condensation structure p-Ph-CH_2_-NHCO- of the para-active group of hydroxymethyl phenol with HBP-NH_2_. The pure PF resin has no peak at this point, and the PFC5 resin has a new peak at this point. It indicates that the addition of HBP-NH_2_ is involved in the synthesis of PF resin. In addition, at 40–41 ppm, the structure corresponds to the para-condensation of p, p-Ph-CH_2_-Ph between phenols or hydroxymethyl phenols. The co-condensation structure o-Ph-CH_2_-NHCO- is also present at this site. It is formed by the co-condensation of hydroxymethyl phenol with HBP-NH_2_ or hydroxymethyl HBP-NH_2_ with phenol. The percentage of integral area at 40–41 ppm was 5.57% for the pure PF resin and 7.01% for the PFC5 resin. The increase in the integral area percentage of PFC5 resin at this site indicates an increase in the co-condensation structure of the modified PF resin, proving that HBP-NH_2_ reacts with the PF resin structure.

**Scheme 1:**
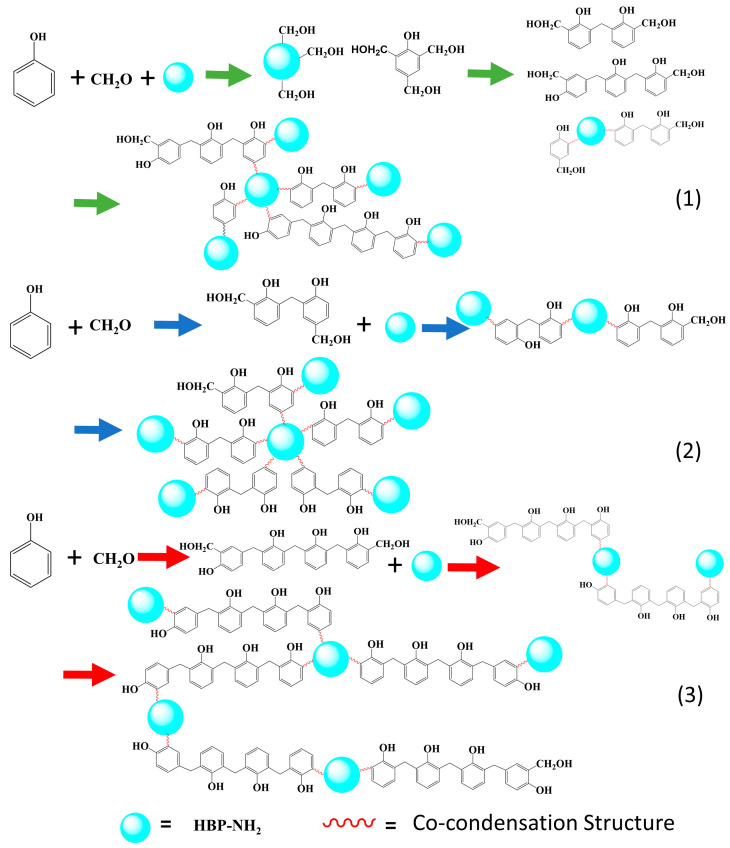
Possible reactions to PFA, PFB, and PFC resins. Note: (1) = The synthesis of PFA, (2) = the synthesis of PFB, (3) the synthesis of PFC.

The terminal group of HBP-NH_2_ is structurally similar to urea. Based on the curing performance test and structural characterization of HBP-NH_2_ modified PF resin, and also combined with the reaction mechanism of PUF, the possible mechanism of the involvement of HBP-NH_2_ in the reaction of PF resin was speculated as shown in Scheme 1.

Scheme 1 shows the schematic diagram of synthesis mechanism when HBP-NH_2_ is added at different synthesis stages of PF resin. The synthesis mechanism of PFA resin is shown in Scheme 1 (1). At the beginning of synthesis, phenol, formaldehyde, and HBP-NH_2_ are added simultaneously. Both phenol and HBP-NH_2_ may undergo hydroxymethylation reactions. There may be two structures in the synthetic resin system, namely, the self-condensation of phenolic resin, and the co-condensation of phenol and HBP-NH_2_. Due to the fact that the system is still dominated by self-polycondensation of phenolic resin, the reduction in curing time is not significant. The cured cross-linked structure is formed by random combination of self-condensation and co-condensation of phenolic resin.

Scheme 1 (2) is a possible synthesis mechanism for PFB resin. HBP-NH_2_ is added after hydroxymethylation of phenol. The amino terminal group of HBP-NH_2_ reacts with hydroxymethylphenol to form a polycondensation structure. Further cross-linking and curing, the highly branched polyurea structure and the phenol ring are cross-linked to form a dense cross-linking network structure, and the resin has a high bonding strength. The test results in Figure 6 also confirm this conclusion. Regardless of the amount of HBP-NH_2_ added, the bonding performance of PFB resin is higher than that of other resins.

The synthesis mechanism of PFC resin is shown in Scheme 1 (3). After a certain time of phenolic resin reaction, HBP-NH_2_ was added. At this point, the phenolic resin in the system forms a certain molecular chain. And then it combines with the highly branched polyurea structure to form a larger molecular structure. This structure has a larger molecular weight, while the larger molecular weight is thought to have a shorter gel time. This result was also confirmed in the GPC and the gel time analysis. Therefore, PFC resins can form a cross-linked network structure rapidly during curing. The network structure is the connection between molecular chain segments and highly branched structures.

## 4. Conclusions

The analysis of gel time, GPC, DSC, DMA, and ^13^C-NMR results showed that the addition of highly branched polyurea HBP-NH_2_ could participate in the synthesis of PF resin and increase its relative molecular weight. It can effectively improve the curing performance of PF resin. And it performed faster curing, shorter gel times, and lower curing temperatures. The plywood performance test results indicated that the addition of HBP-NH_2_ after hydroxymethylation reaction of phenol and formaldehyde could enhance the bonding performance of PF resin. Therefore, reasonable control of the HBP-NH_2_ addition process could not only improve the curing rate of PF resin, but also improve the bonding performance. The comprehensive comparison test results showed that PFC5 resin has good curing performance, which means adding 5% HBP-NH_2_ to prepare PF resin 1.5 h before the end of PF resin synthesis. Finally, the possible mechanism of HBP-NH_2_ modified PF resin was briefly introduced.

## Figures and Tables

**Table 1:** The basic properties of resins under different synthesis conditions.

Resin	Solid Content (%)	Viscosity (MPa·s)	Storage Period (Days)	Gel Time at 110 °C (s)	Gel Time at 130 °C (s)
PF	46.19 (±0.21)	414 (±5)	<60	592 (±21)	445 (±05)
PFA1	46.60 (±0.15)	426 (±6)	>60	584 (±20)	367 (±10)
PFA3	46.97 (±0.23)	446 (±8)	>60	497 (±18)	364 (±08)
PFA5	46.85 (±0.19)	533 (±3)	>60	481 (±24)	358 (±10)
PFA7	46.16 (±0.17)	669 (±1)	>60	446 (±17)	347 (±11)
PFA9	44.99 (±0.26)	835 (±4)	>60	440 (±21)	383 (±19)
PFA11	44.89 (±0.15)	994 (±5)	>60	472 (±18)	403 (±17)
PFB1	46.06 (±0.20)	469 (±3)	>60	588 (±16)	377 (±18)
PFB3	45.85 (±0.22)	492 (±2)	>60	458 (±19)	373 (±11)
PFB5	45.64 (±0.19)	575 (±2)	>60	447 (±15)	311 (±10)
PFB7	46.25 (±0.18)	780 (±4)	>60	450 (±20)	343 (±17)
PFB9	46.63 (±0.20)	1037 (±7)	>60	468 (±19)	321 (±15)
PFB11	47.71 (±0.16)	1209 (±2)	>60	448 (±20)	304 (±17)
PFC1	46.90 (±0.14)	268 (±3)	>60	580 (±18)	335 (±10)
PFC3	46.10 (±0.18)	283 (±2)	>60	404 (±14)	219 (±08)
PFC5	46.40 (±0.15)	365 (±2)	>60	401 (±12)	226 (±08)
PFC7	46.66 (±0.23)	513 (±1)	>60	460 (±20)	340 (±05)
PFC9	47.82 (±0.12)	816 (±5)	>60	456 (±19)	328 (±14)
PFC11	46.74 (±0.20)	877 (±6)	>60	480 (±16)	332 (±15)

**Table 2:** The GPC results of resins under different synthesis conditions.

Resin	Mn	Mw	PDI
PF	11,918	18,005	1.51
PFA5	12,167	20,817	1.71
PFB5	13,459	21,832	1.62
PFC5	15,012	23,821	1.59

**Table 3:** The^13^C-NMR peak attribution of phenol-formaldehyde resins.

Structure	Chemical Shifts (ppm)	Integral Area Share (%)	
		PF	PFC5
o,o-Ph-CH_2_-Ph	29–31	88.38	86.73
o,p-Ph-CH_2_-Ph	34–36	6.05	6.20
p,p-Ph-CH_2_-Ph o-Ph-CH_2_-NHCO-	40–41	5.57	7.01
p-Ph-CH_2_-NHCO-	45–46	0.00	0.06
Total		100	100

## Data Availability

Not applicable.
